# The transcription factor ccaat/enhancer binding protein β (*C/EBPβ*) and miR-27a regulate the expression of porcine Dickkopf2 (*DKK2*)

**DOI:** 10.1038/srep17972

**Published:** 2015-12-11

**Authors:** Hu Tao, Lei Wang, Jiawei Zhou, Panfei Pang, Shanzhi Cai, Jialian Li, Shuqi Mei, Fenge Li

**Affiliations:** 1 Key Laboratory of Pig Genetics and Breeding of Ministry of Agriculture & Key Laboratory of Agricultural Animal Genetics, Breeding and Reproduction of Ministry of Education, Huazhong Agricultural University, Wuhan 430070, PR China; 2 Hubei Key Laboratory of Animal Embryo Engineering and Molecular Breeding, Hubei Academy of Agriculture Science, Wuhan, 430064, PR China; 3 The Cooperative Innovation Center for Sustainable Pig Production, Wuhan 430070, PR China

## Abstract

Using Affymetrix porcine Gene-Chip analyses, we found that Dickkopf2 (*DKK2*), a WNT antagonist, is differentially expressed in pre-ovulatory follicles between Large White and Chinese Taihu sows. This study aims to identify the regulatory factors responsible for *DKK2* expression. Deletion fragment and mutation analyses identified *DKK2-D3* as the porcine *DKK2* core promoter. There were four C/EBPβ binding sites within the *DKK2* core promoter. The C allele that results from a spontaneous alteration (*DKK2* c.−1130 T > C) in the core promoter was associated with a higher total number born (TNB) and a higher number born alive (NBA) in all parities in a synthetic pig population. This was possibly the result of a change in C/EBPβ binding ability, which was confirmed using chromatin immunoprecipitation (ChIP) and electrophoretic mobility shift assays (EMSA). Moreover, C/EBPβ specifically bound to and activated the *DKK2* promoter, as revealed by mutation analysis, overexpression and RNA interference (RNAi) experiments. We also confirmed that miR-27a is a negative regulator of the *DKK2* gene using miR-27a overexpression and inhibition experiments and mutation analyses. RTCA xCELLigence experiments showed that miR-27a suppressed Chinese hamster ovary (CHO) cell proliferation by down-regulating *DKK2* gene expression. Taken together, our findings suggest that C/EBPβ and miR-27a control *DKK2* transcription.

Mammalian folliculogenesis is a complex process through which primordial follicles develop into pre-ovulatory follicles. It is followed by ovulation, which releases mature oocytes^1^. After ovulation, the remaining follicular structure undergoes luteinization, and the former granulosa and thecal cells are transformed into follicular and thecal lutein cells. The complexity of folliculogenesis indicates that tightly regulated gene expression and interactions between many genes are required for successful oocyte development. Approximately 100 genes have been shown to be essential for normal folliculogenesis in mice in knock-out experiments^2^.

WNT signaling proteins have been shown to play crucial roles in reproductive processes, including foetal development, ovarian development, gestation and mammogenesis^3^,^4^,^5^. Six WNT/β-catenin signaling pathway genes, including the wingless-type MMTV integration site family member 10B (*WNT10B*) and *DKK2*, have differential expression patterns in PMSG-hCG-stimulated pre-ovulatory ovarian follicles in three Chinese Taihu and three Large White cycling sows, as shown by Affymetrix Porcine GeneChip™ analyses^6^. *DKK2* is a direct inhibitor of WNT binding to LDL receptor-related proteins 5/6 (LRP5/6), which are co-receptors of frizzled^7^,^8^. The role of *DKK2* in tumourigenesis and WNT signaling has been partially described^9^,^10^,^11^, but no direct evidence for its functions in follicle development have yet been reported. After studying the literature, we hypothesized that *DKK2* functions may be associated with embryo implantation and endometrial membrane stripping^8^.

Recent studies have shown that miRNAs functions in cell invasion and tumourigenesis involve the WNT/β-catenin signaling pathway, including *DKK2*. For example, miR-21 promotes oral tongue squamous cell carcinoma (OTSCC) invasion via the WNT/β-catenin pathway by targeting *DKK2 in vitro*^12^. MiR-1260b binds to the 3′ untranslated regions (UTRs) of the *DKK2*, secreted frizzled-related protein 1 (*sFRP1*) and SMAD family member 4 (*Smad4*) mRNAs, thereby down-regulating their expression in renal cancer cells^11^. MiR-222 promotes tumourigenesis by targeting *DKK2* and activating the WNT/β-catenin signaling pathway^10^.

The objective of this study was to examine mutations and cis regulatory elements in the porcine *DKK2* gene. We therefore analysed the 5′ upstream sequences and 3′ UTR of *DKK2* to better understand its role.

## Results

### Identification and characterization of the porcine *DKK2* promoter

**Figure 1:**
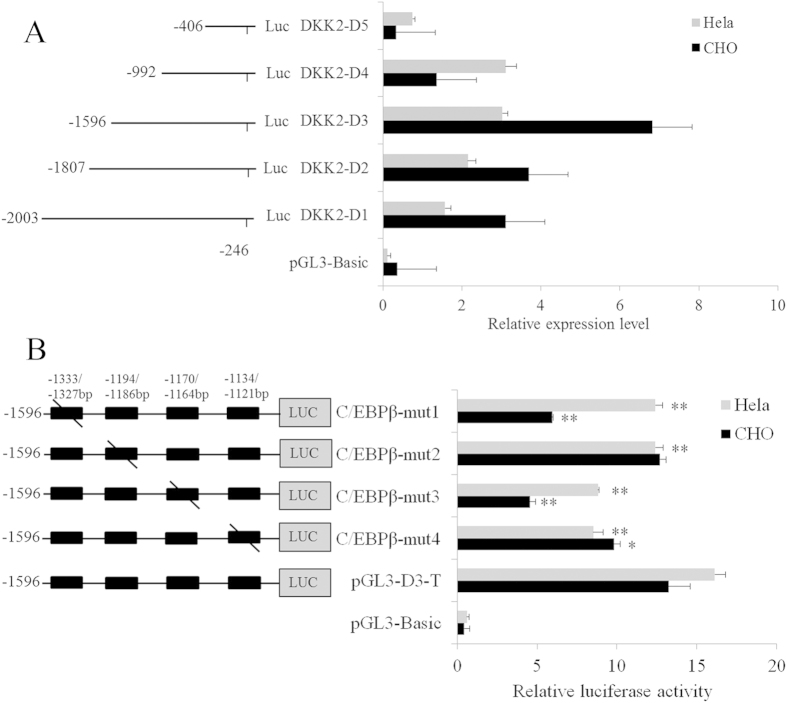
Site-directed mutagenesis and 5′-deletion analysis of C/EBPβ binding sites in the *DKK2* promoter. (**A**) Promoter activity was analysed in a series of deleted constructs using luciferase assays. Left panel, schematic representation of the mutants linked with the luciferase gene in the pGL3 vector. The nucleotides are numbered from the potential transcription start site, which was assigned +1. Right panel, the relative activity levels of a series of *pGL3-D* mutant constructs were determined using luciferase assays. (**B**) Point mutation analysis in the CCAAT boxes of the *DKK2* promoter were analysed using luciferase assays. Left panel, schematic structure of site-directed mutagenesis in the putative C/EBPβ binding sites of the *DKK2* gene. Right panel, site-directed mutagenesis in the C/EBPβ binding sites of the *DKK2* promoter was analysed using luciferase assays. Non-modified *pGL3* was used as a negative control. Firefly luciferase activity was normalized to *Renilla* luciferase activity, and the data are shown as the fold increase/decrease over the luciferase activity observed for *pGL3-D3* in CHO and HeLa cells. The data are expressed as the mean ± SE of three replicates. *P < 0.05 and **P < 0.01 compared to the *pGL3-D3* construct.

To identify the promoter region and regulatory elements of the porcine *DKK2* gene, we used luciferase assays to analyse a series of deletions in the potential promoter region predicted by neural network promoter prediction online software. Luciferase activity analysis in both CHO and the Henrietta Lacks strain of cervical cancer cells (HeLa cells) revealed that *DKK2*-D3 (−1596 bp/−992 bp) was the potential core promoter region that is important for *DKK2* transcription (Fig. 1A). Four C/EBPβ transcription factor binding sites (−1333 bp/−1327 bp, −1194 bp/−1186 bp, −1170 bp/−1164 bp and −1134 bp/−1121 bp) were identified in the *DKK2*-D3 region using the transcription factor prediction software TFsearch 1.3 (Fig. 1B). Four *C/EBPβ* mutants (*C/EBPβ-mut1*, *mut2, mut3* and *mut4*), which were introduced using site mutagenesis (Fig. 1B), were transfected into CHO and HeLa cells. *C/EBPβ-mut1*, *mut3* and *mut4* significantly decreased promoter activity in CHO cells (*P* < 0.05) (Fig. 1B). Promoter activity was markedly reduced by all four *C/EBPβ* mutants in HeLa cells (*P* < 0.01) (Fig. 1B). These results indicate that *DKK2*-D3 (−1596 bp/−992 bp) is the potential core promoter region and that the four C/EBPβ binding sites are important for *DKK2* promoter activity.

### A spontaneous T/C mutation in the *DKK2* 5′ flanking sequence affects promoter activity

**Figure 2:**
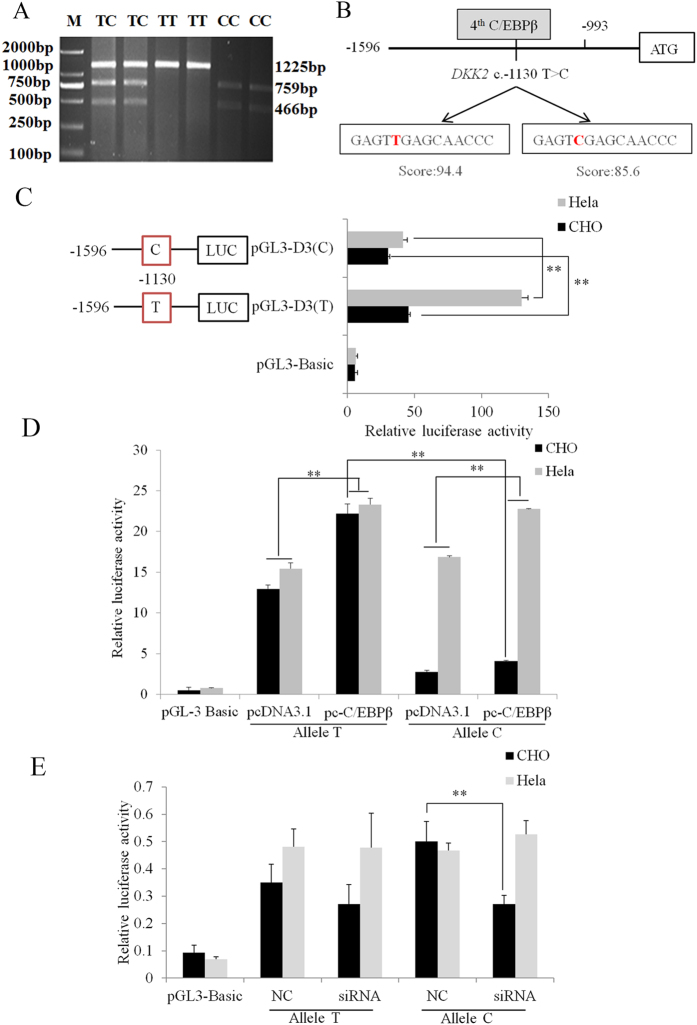
The T/C mutation in the *DKK2* 5′ flanking sequence *DKK2* c.−1130 T > C affected promoter activity in CHO and HeLa cells. (**A**) Genotyping for *DKK2* c.−1130 T > C using PCR-*Taq*I-RFLP. Genotype TC: 1225 bp+759 bp+466 bp; Genotype TT: 1225 bp; Genotype CC: 759 bp+466 bp. M: DNA molecular marker DL 2,000. (**B**) Schematic structure of the *DKK2* c.−1130 T > C mutation in the fourth C/EBPβ binding site. (**C**) *pGL3-D3-T* and *pGL3-D3-C* were transfected into CHO and HeLa cells. Compared to *pGL3-D3-C*, *pGL3-D3-T* transfections resulted in significantly higher relative luciferase activity (*P* < 0.01). (**D**) *pGL3-D3-T* and *pGL3-D3-C* were co-transfected with the *pGL3-*C/EBPβ expression plasmid, resulting in increased *DKK2* promoter luciferase activity. In the *pc-C/EBPβ* groups, the T allele group showed a significantly higher level of relative luciferase activity than the C allele group in CHO cells (*P* < 0.01). (**E**) siRNA (2 μl) was co-transfected with 0.2 μg *pGL3-D3-T* or *pGL3-D3-C* for 24 h in CHO and HeLa cells, and *DKK2* promoter luciferase activity was inhibited. The *pGL3-basic* plasmid was used as a negative control. The *pRT-TK* plasmid served as an inner control during analysis of transfection efficiency. **P < 0.01.

One spontaneous T/C mutation, *DKK2* c.−1130 T > C in the core promoter region (−1596 bp/−992 bp), was detected by comparative sequencing in Large White and Chinese Meishan pigs (Fig. 2A). The T allele was fixed in Large White, Duroc and Landrace pigs (Supplementary Table S1). Association analyses were performed in pigs from a synthetic pig line -the 4th dam line of Chinese lean-type new line (DIV), and the results showed that TC pigs had higher NBA (1.11 and 1.58) than was observed in TT and CC pigs (*P* < 0.05) in all parities, and had higher TNB (1.23) than was observed in TT pigs (*P* < 0.05) in all parities (Supplementary Table S2). Therefore, *DKK2* c.−1130 T > C was found to be associated with TNB and NBA in all parities in DIV pigs.

The *DKK2* c.−1130 T > C mutation changed the binding ability at the fourth C/EBPβ binding site (−1134 bp/−1121 bp) as revealed by the TFsearch scores for C/EBPβ at the T allele (94.4) and the C allele (85.6) (*P* < 0.05 by chi-square test) (Fig. 2B). The *pGL3-D3-T* and *pGL3-D3-C* plasmids were transfected into CHO and HeLa cells. *pGL3-D3-T* showed a significantly higher relative luciferase activity than *pGL3-D3-C* (*P* < 0.01) (Fig. 2C). The porcine C/EBPβ cDNA (NM_001199889.1) was then cloned into *pcDNA3.1* (Invitrogen). The *pc-C/EBPβ* and *pcDNA3.1* plasmids were co-transfected into CHO and HeLa cells with *pGL3-D3-T* and *pGL3-D3-C*. The relative luciferase activity of both *pGL3-D3-T* and *pGL3-D3-C* in the *C/EBPβ* overexpressing groups were significantly elevated compared to the control group (*P* < 0.01), and the T allele group showed significantly higher relative luciferase activity than the C allele group in CHO cells (*P* < 0.01) (Fig. 2D). To our knowledge, we are the first group to discover the effects of two different alleles at *DKK2 c.−1130 T* > *C* on the transcription of the *DKK2* gene. In the C allele group, CHO cells transfected with small interfering RNAs (siRNAs) showed nearly 2-fold higher luciferase activity than the control group (*P* < 0.01) (Fig. 2E). Thus, the activity of the *DKK2* promoter at the *c*.−*1130 T* allele was significantly enhanced compared to activity at the *c*.−*1130* C allele.

### *C/EBPβ* regulates *DKK2* gene expression, as shown in overexpression and RNA interference experiments

**Figure 3:**
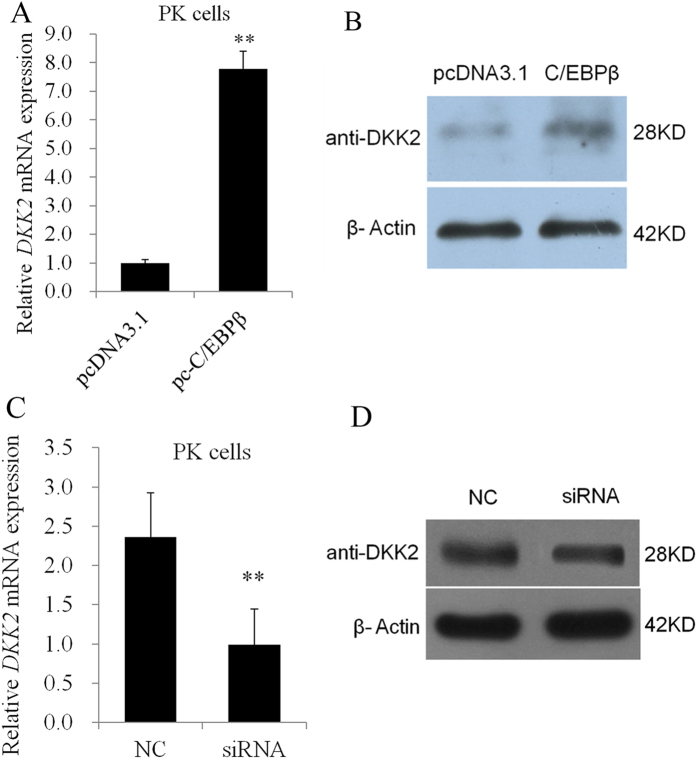
C/EBPβ promotes the transcription of the porcine *DKK2* gene. (**A**) Overexpression of C/EBPβ stimulated *DKK2* mRNA expression in PK cells. (**B**) C/EBPβ was transfected into PK cells, and transfection efficiency was determined using Western blot analysis. (**C**) Knockdown of C/EBPβ was confirmed using qRT-PCR analysis. PK cells were stimulated using siRNA or NC (10 μl) for 24 h. (**D**) The expression of the DKK2 protein was determined using Western blot analysis. **P < 0.01.

Overexpressing *C/EBPβ* in pig kidney (PK) cells significantly promoted *DKK2* expression, as shown using quantitative real time PCR (qRT-PCR) (Fig. 3A) and Western blot analysis (Fig. 3B) (P < 0.01). Inhibiting *C/EBPβ* expression significantly suppressed *DKK2* expression at both the mRNA (Fig. 3C) and the protein level (Fig. 3D, Supplementary Fig. S1) (*P* < 0.01). These findings suggest that *C/EBPβ* plays an essential role in *DKK2* expression.

### The transcription factor C/EBPβ binds to the *DKK2* promoter *in vitro* and *in vivo*

**Figure 4:**
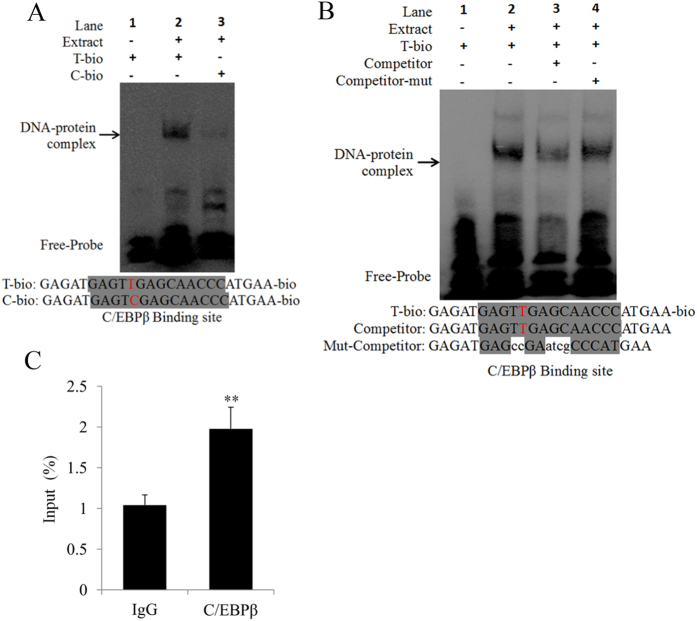
Binding of C/EBPβ with the *DKK2* promoter was analysed using EMSA and ChIP assays. (**A**) Probes were incubated with nuclear extracts in the presence of T-bio and C-bio probes. Specific DNA-protein complex bands are indicated by arrows. The probe sequences are shown under the panel. (**B**) The probes were incubated with nuclear extracts in the absence or presence of a 50-fold excess of various competitor probes (mutant or non-labelled probes). The specific DNA-protein complex bands are indicated by arrows. The sequences of the various probes are shown under the panel. (**C**) ChIP assay to analyse C/EBPβ binding to the *DKK2* promoter in PK cells. The interaction of C/EBPβ *in vivo* with the *DKK2* promoter was analysed using ChIP analysis. DNA isolated from immunoprecipitated materials was amplified using qRT-PCR. Total chromatin was used as the input. Normal mouse IgG was used as the negative control.

To examine the effects of *DKK2* c.−1130 T > C on the ability of C/EBPβ to bind to the promoter, a competitive EMSA was performed. For EMSA, we used biotin-labelled T allele (T-bio) and C allele (C-bio) oligonucleotides that spanned the fourth C/EBPβ element of the *DKK2* D3 promoter. Nuclear extracts were isolated from pig ovarian follicles. The results revealed that nuclear extracts with the T-bio probe contained DNA-protein complexes, while only a weak band was observed in C-bio probe extracts (Fig. 4A). The T-bio probe displayed significantly stronger binding activity, and this was confirmed using competitive and mut-competitive probes (Fig. 4B). Thus, our data indicate that the *DKK2* c.-1130 T > C within the fourth C/EBPβ binding region of the *DKK2* core promoter determines the binding affinity of C/EBPβ to the *DKK2* promoter.

ChIP analysis was performed in PK cells to determine whether C/EBPβ can bind to the *DKK2* promoter *in vivo*. Soluble chromatin was immunoprecipitated using a C/EBPβ antibody against DNA binding proteins. qRT-PCR was performed using primers specific to the C/EBPβ binding site in the *DKK2* promoter (Supplementary Table S3). The ChIP assay showed that C/EBPβ interacted with the *DKK2* promoter within the binding site (Fig. 4C). These results confirm that the transcription factor C/EBPβ is capable of binding to the fourth C/EBPβ binding site in the *DKK2* promoter region *in vivo*.

### C/EBPβ regulates WNT/β-catenin signaling pathway gene expression via the *DKK2* gene

**Figure 5:**
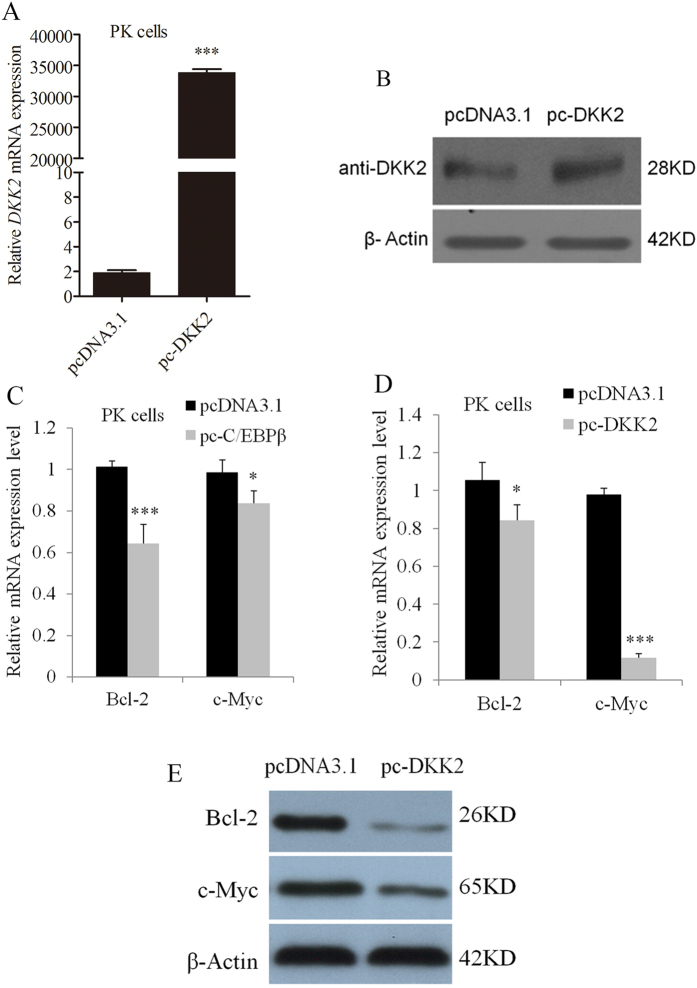
Overexpressing *DKK2* and *C/EBPβ* repressed the mRNA expression levels of *Bcl2* and *c-Myc.* (**A**) The *pc-DKK2* vector was transfected into PK cells for 24 h, resulting in increased *DKK2* mRNA and (**B**) protein expression levels. (**C**) The mRNA expression levels of *Bcl2* and *c-Myc* were down-regulated when cells were transfected with the *pc-C/EBPβ* vector or (**D**) the *pc-DKK2* vector. (**E**) The protein expression levels of *Bcl2* and *c-Myc* were down-regulated when cells were transfected with the *pc-DKK2* vector. *P < 0.05; **P < 0.01; ***P < 0.001.

We hypothesized that C/EBPβ might play a role in WNT/β-catenin signaling via its effects on *DKK2* gene expression. First, the porcine *DKK2* cDNA (XM_003129269.2) was cloned into *pcDNA3.1* (Invitrogen). The *pc-DKK2* plasmid was transfected into PK cells. *DKK2* overexpression significantly promoted *DKK2* expression, as determined using qRT-PCR and Western blot analysis (*P* < 0.01) (Fig. 5A,B), indicating that the recombinant plasmid in *pc-DKK2* was successfully expressed. Furthermore, overexpression of *C/EBPβ* repressed the mRNA expression levels of B-Cell CLL/Lymphoma 2 (*Bcl2*) and a cellular oncogene (*c-Myc*) compared to controls (Fig. 5C). Overexpression of *DKK2* repressed both the mRNA and protein expression levels of *Bcl2* and *c-Myc*, which are downstream targets in the WNT/β-catenin signaling pathway (Fig. 5D,E). These results suggest that C/EBPβ inhibits WNT/β-catenin signaling via the *DKK2* gene.

### *DKK2* is a direct target of miR-27a

Recent studies have demonstrated that miRNAs may play pivotal roles in regulating the expression of genes that are essential for ovarian folliculogenesis and endocrine function in mice^2^. For example, miR-224 was reported to control mouse granulosa cell proliferation and functions by targeting *Smad4*^13^.

**Figure 6:**
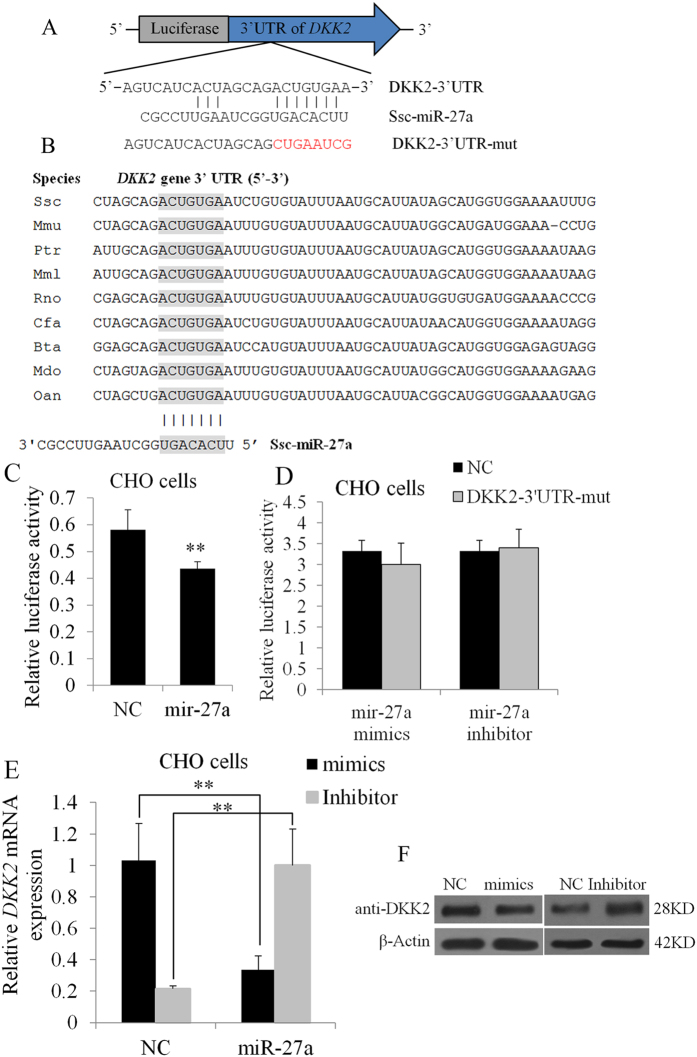
*DKK2* is a target of miR-27a. (**A**) Binding sites for miR-27a in the *DKK2* 3′UTR were predicted using TargetScan and RNAhybrid software. Red font indicates sequences that were mutated to abolish the interaction between miR-27a and the *DKK2* 3′UTR. (**B**) The miR-27a binding site sequences in the *DKK2* 3′UTR in different species. The miRNA binding sites are shown in grey. *Mmu, Mus musculus; Ptr, Pan troglodytes; Mml, Macaca mulatta; Rno, Rattus norvegicus*; *Cfa, Canis familiaris; Bta, Bos taurus; mdo, Monodelphis domestica;* and *Oan, Ornithorhynchus anatinus.* (**C**) *pmirGLO-DKK2* was co-transfected into CHO cells with miR-27a mimics or NC. Whole cellular lysates were obtained 24 h after transfection, and relative luciferase activity was then measured. (**D**) *pmirGLO-DKK2-mut* was transfected into CHO cells with miR-27a mimics or NC. (**E**) Endogenous *DKK2* mRNA levels were detected in CHO cells 24 h after transfection with miR-27a mimics or NC mimics and inhibitor or inhibitor NCs. (F) DKK2 protein levels were also monitored using Western blot analysis for 48 h after transfection with miR-27a mimics or mimic NC and inhibitor or inhibitor NCs. **P < 0.01.

To determine whether a miRNA regulates *DKK2* expression, we identified miRNAs that might bind to the 3′ UTR of the *DKK2* gene. TargetScan and RNAhybrid software predicted that the *DKK2* 3′ UTR contains a putative binding site for miR-27a (Fig. 6A). In addition, the miR-27a binding seed sequence in the *DKK2* 3′UTR is highly conserved in mammals (Fig. 6B). To determine whether *DKK2* is a direct target of miR-27a, the *pmirGLO-DKK2* luciferase reporter was co-transfected with miR-27a mimics or a negative control (NC) into CHO cells. As shown in Fig. 6B, overexpression of miR-27a reduced the luciferase activity of the *pmirGLO-DKK2* reporter. To further understand the specificity of this miR-27a-*DKK2* binding site, we constructed mutated *pmirGLO-DKK2-mut* luciferase reporters (Fig. 6A). We discovered that these mutations completely abolished the suppression of luciferase activity observed in the wild-type vector (Fig. 6D). MiR-27a dramatically suppressed endogenous *DKK2* expression in CHO cells (Fig. 6E,F). Moreover, inducing the reduction of miR-27a expression using a miR-27a inhibitor led to an increase in *DKK2* expression (Fig. 6E,F). These findings indicate that *DKK2* is a direct target of miR-27a.

### xCELLigence system-based real-time monitoring of CHO cell proliferation

**Figure 7:**
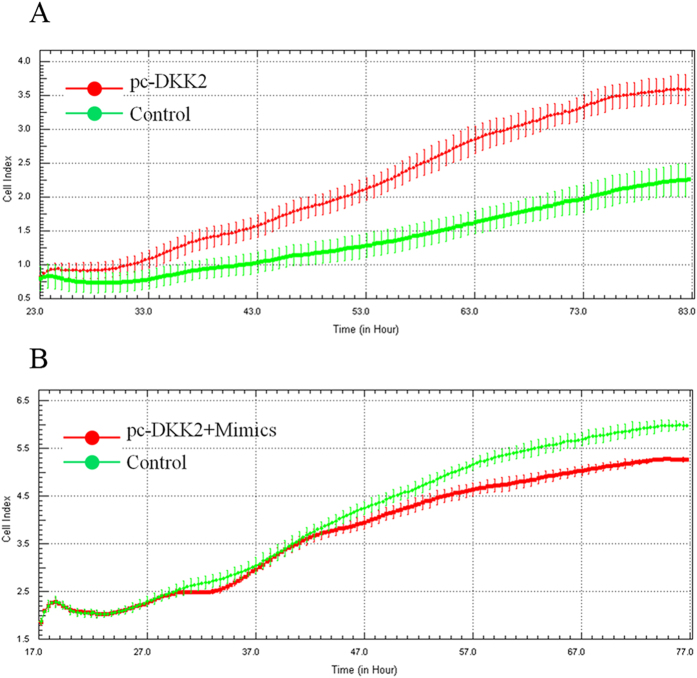
CHO cells were transfected with pooled miR-27a mimics or NC when the cell index reached 1.0, and cell growth dynamics were then continuously monitored using an xCELLigence system.

To determine the effects of miR-27a and *DKK2* on cell proliferation, CHO cells were transfected with *pc-DKK2* or *pcDNA3.1* plasmids and pooled miR-27a mimics or a NC when the cell index reached 1.0. Then, cell growth dynamics were continuously monitored using the xCELLigence system. *pc-DKK2*-transfected cells displayed an increased growth rate compared to control cells at 24 h post-transfection (Fig. 7A), while miR-27a mimics co-transfected with *pc-DKK2* cells displayed a reduced growth rate compared to control cells (Fig. 7B). These results suggest that miR-27a inhibits the proliferation of CHO cells by targeting *DKK2*.

## Discussion

Follicular development is a complex biological process. However, the regulatory mechanism underlying follicular development remains unclear. The WNT signaling pathway has been demonstrated to regulate cell-cell interactions during embryogenesis and follicle maturation by controlling steroidogenesis in postnatal ovaries in mammals^14^,^15^. More recent evidence has begun to provide data on the importance of WNT components, including WNT2, WNT3a and WNT4, in adult ovarian functions related to follicle development^16^. The *DKK2* gene, the key inhibitory factor of WNT/β-catenin signaling pathway, was differentially expressed in the PMSG-hCG stimulated pre-ovulatory ovarian follicles of Chinese Taihu and Large White sows and was located to quantitative trait locus (QTL) intervals for litter traits^6^. *DKK2* gene was expressed in P0-21 ovaries, but not in the full-grown oocyte, ovulated oocyte and embryos, suggesting *DKK2* was expressed in ovarian granulosa cells^17^.

We isolated the 5′ flanking region of the porcine *DKK2* gene and analyzed its promoter region and regulatory elements. The *DKK2-*D3 (−1596 bp/−992 bp) region had the maximal promoter activity revealed by the luciferase reporter analysis (Fig. 1A). C/EBPβ, a notable member of C/EBPs family, affected a series of important reproduction processes including uterine stromal differentiation and embryo implantation^18^, female fertility and decidualization^19^. We found C/EBPβ regulated *DKK2* transcription by binding to the *DKK2* core promoter (*DKK2-*D3) by site-directed mutagenesis, EMSA and *C/EBPβ* overexpression.

In the present research, *DKK2* c.−1130 T > C located in the core promoter region was associated with TNB and NBA of all parities in DIV pigs, suggesting *DKK2* gene may be the potential candidate gene for QTL for uterine capacity at 71 cM (53–107 cM)^20^, QTL for teat number at 73.5 cM (56.1–96.3 cM)^21^ and QTL for numbers of corpus luteum at 107.5 cM (59.3–107.5 cM)^22^. Allele T frequency in all Western breeds (Large White, Pietrain, Duroc, White Duroc and Landrace) was 1, while the C allele was the dominant allele in Taihu pigs, Huainan pigs and Hezuo pigs. We suspect that homozygous C alleles may lead to embryonic death during early development.

*DKK2* c.−1130 T > C mutation may change the binding ability for the fourth C/EBPβ binding site (−1134 bp/−1121 bp), with the TFsearch score of C/EBPβ 94.4 for allele T and 85.6 for allele C (*P* < 0.05 by Chi-square test). We further found *DKK2* with *c*.−*1130 T* promoter activity was significantly enhanced compared to allele *c*.−*1130* C in luciferase activity assays. Above all, *DKK2* c.−1130 T > C may affect the litter size by changing the *DKK2* gene expression levels via the binding of the transcription factor C/EBPβ.

G3072A mutation in the intron 3 of insulin-like growth factor 2 (*IGF2*), a well-known pig quantitative trait nucleotide (QTN) for muscle growth, occurs in an evolutionarily conserved CpG island that is hypomethylated in skeletal muscle. The wild-type G nucleotide at *IGF2*-intron3–3072 binds a nuclear factor zinc finger, BED-type containing 6 (*ZBED6*), but this interaction is abrogated by the mutation or methylation of the actual CpG site^23^. *ZBED6* mediates *IGF2* gene expression by regulating promoter activity and DNA methylation in myoblasts^24^. The callipyge mutation in sheep enhances the expression of co-regulated imprinted genes in *cis* without affecting their imprinting status, which shares some similar features with the *IGF2* mutation in pigs^25^,^26^.

*DKK2* expression was epigenetically silenced by methylation in renal cell carcinoma (RCC) and the methylation frequency of the *DKK2* gene promoter region is higher in RCC patients with higher grades and stages of the disease^7^. The spontaneous mutation *DKK2* c.−1130 T > C happened to be located in a CpG site in the *DKK2* promoter. To determine the methylation status of this CpG site, we performed a bisulfite sequencing of the genomic DNA isolated from a CC Tongcheng pig. The results showed that this CpG site was completely un-methylated, indicating that the methylation levels have no effect on the binding activities of C/EBPβ (Supplementary Fig. S2). Therefore, *DKK2* c.−1130 T > C led to changes in the C/EBPβ binding abilities but not in the DNA methylation status.

MiRNAs are another type of regulatory factors that may play pivotal roles in regulating the expression of genes essential for ovarian folliculogenesis and endocrine function in mice^2^. MiR-224 has been shown to control mouse granulosa cell proliferation and function by targeting *Smad4*^13^. MiR-27a was a differentially expressed miRNA in the pre-ovulatory follicles from Large White and Chinese Taihu sows^27^. The relative expression of *mus musculus* (*mmu*) miR-27a (0.57 ± 0.18, *P* = 0.016) was significantly lower in the GCs of follicles containing MII oocytes compared with those of MI oocytes 27. Transfection with a mmu-miR-27a-mimic sequence decreased the oocyte maturation rate compared with that of the control (9.4 versus 18.9%, *P* = 0.042), and transfection with mmu-miR-27a inhibitor sequences increased the oocyte maturation rate by 1.67-folds compared with that of the control (31.6% versus 18.9%, *P* = 0.013)^28^. A previous study of miR-27a showed that the down-regulation of miR-27a inhibited the proliferation of gastric cancer cells^29^.

Two downstream genes of WNT/β-catenin signaling pathway (*Bcl2* and *c-Myc)* expressions were detected due to their relations with folliculogenesis. *Bcl2,* a marker gene for apoptosis, was considered primary executioners and regulators of ovarian follicle death or survival, and was a pro-survival factor expressed in the developing follicles^30^,^31^. *c-Myc* could promote the proliferation of granulosa cells and in the regulation of proliferative events involved in luteal formation^32^,^33^. Expressions of the downstream genes of WNT/β-catenin signaling pathway, including *β-catenin*, *c-Myc* and *cyclin D1*, were decreased in *DKK2*-transfected cells compared with mock cells^9^. The inhibited *DKK2* expression lead to up-regulation of the protein levels of *Bcl2* and *c-Myc* in glioma cells^10^. Thus, we choose the downstream genes of WNT/β-catenin signaling pathway *Bcl2* and *c-Myc* as the target genes for folliculogenesis.

**Figure 8:**
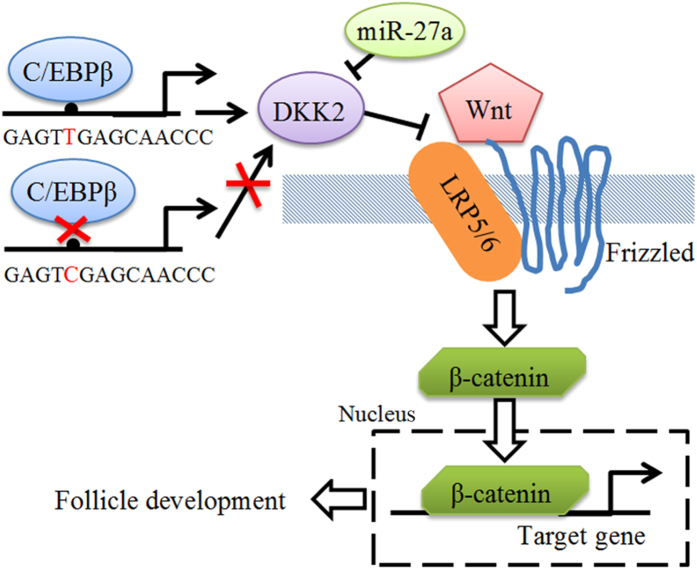
A graphical abstract showing the main findings of this study. The *DKK2* gene is regulated by the transcription factor C/EBPβ and miR-27a, and this process likely contributes to follicle development by inhibiting canonical WNT/β-catenin signaling.

*DKK2* was reported to be associated with the embryo implantation^8^. It could be synthesized in the surrounding granulosa cells and transported to the oocytes^17^. Luciferase reporter assay in human ovarian cancer cells including SKOV3 and ES-2 cells showed that *TCF* activity was significantly suppressed in *DKK2*-transfected cells^9^. Consistent with the above reports, overexpressions of *C/EBPβ* and *DKK2* could repress the mRNA expression levels of *Bcl2* and *c-Myc*, two downstream genes of the WNT/β-catenin signaling pathway (Fig. 5C–E), indicating that C/EBPβ acted as an inhibitor of the WNT/β-catenin via the *DKK2* gene. Therefore, we postulated *DKK2* c.−1130 T > C might affect C/EBPβ binding to the *DKK2* promoter region, and thus regulate the expression of the *DKK2* to influence follicular development in the WNT/β-catenin signaling pathway (Fig. 8). Our present study is a good complementary to the WNT/β-catenin signaling pathway research.

In summary, the *DKK2* c.−1130 T > C affected C/EBPβ binding ability with the *DKK2* promoter. And the *DKK2* promoter activity was significantly enhanced with allele T of *DKK2* c.−1130 T > C compared with allele C. Our findings strongly suggest that WNT/β-catenin signaling pathway genes are regulated by C/EBPβ and miR-27a via *DKK2*.

## Materials and Methods

### Animals and tissues

All animal procedures were performed according to protocols approved by the Biological Studies Animal Care and Use Committee of Hubei Province, PR China. *DKK2* allele frequency was investigated in 10 pig populations, including 274 Large White pigs, 31 Pietrain pigs, 29 Duroc, 30 White Duroc pigs, 21 Landrace pigs, 129 DIV line pigs, 70 Taihu pigs, 20 Huainan pigs, 37 Tongcheng pigs and 7 Hezuo pigs. Association analyses were conducted in DIV and Large White pigs with TNB and NBA records in 491 litters of DIV sows and in 495 litters of Large White sows. All of the studies involving animals were conducted according to the regulation (No. 5 proclamation of the Standing Committee of Hubei People’s Congress) approved by the Standing Committee of Hubei People’s Congress, PR China. The sample collection was approved by the Ethics Committee of Huazhong Agricultural University with the permit number No. 30700571 for this study. The animals were allowed access to feed and water *ad libitum* under the same normal conditions and were humanely sacrificed as necessary to ameliorate suffering. The methods were carried out in accordance with the approved guidelines.

### *DKK2* gene sequence analysis

Based on the *sus scrofa DKK2* gene sequence (NC_010450.3), primers DKK2-PF/PR and DKK2-PF1/PR1 (Supplementary Table S3) were designed to amplify the 5′ and 3′ stream sequences. The sequences of 3 Large White pigs and 3 Taihu pigs were aligned by using Clustalw2 (http://www.ebi.ac.uk/Tools/msa/clustalw2/) to detect the spontaneous mutations. The potential promoter was analyzed using the online neural network promoter prediction (NNPP) software (http://www.fruitfly.org/seq_tools/promoter.html). Transcription factor binding sites were predicted using the TFSEARCH: Searching Transcription Factor Binding Sites (ver 1.3) (http://www.cbrc.jp/research/db/TFSEARCH.html) with a threshold score of 85. The potential target sites of miRNAs were predicted by TargetScan (http://www.targetscan. org/) and RNAhybrid (http://bibiserv.techfak.uni-bielefeld.de/rnahybrid/).

### Plasmid construction, cell culture, and dual-luciferase reporter assay

Five *DKK2* promoter deletion fragments were amplified using primers D1-D5 (Supplementary Table S3). The purified PCR products were cloned into the *pMD18-T* vector (TaKaRa). Then the recombinant plasmids were digested with *Nhe*I and *Hin*dIII (Thermo), and ligated into the *pGL3*-Basic vector (Promega).

The CHO, HeLa cells and PK obtained from China Center for Type Culture Collection (CCTCC) were cultured as previously described^34^,^35^. Cells were seeded at a density of 1.5 × 10^5^/ml using Dulbecco minimum essential medium (DMEM) supplied with 10% fetal bovine serum (FBS) medium (Gibco). After 18–24 h, cells were transfected using lipofectamine 2000 transfection reagent (Life Technologies). The mutants of binding sites were generated using a MutanBEST Kit (TaKaRa) and mutagenic primers (Supplementary Table S3). Plasmid DNA of each well used in the transfection containing 0.8 μg of the *DKK2* promoter or 3′UTR constructs and 0.04 μg of the internal control vector PRL-TK Renilla/luciferase plasmid. The enzymatic activity of luciferase was then measured with PerkinElmer 2030 Multilabel Reader (PerkinElmer).

### Overexpression and inhibition

One day before transfection, cells were plated in growth medium without antibiotics. Cells were transfected with miRNA mimics, inhibitors and NC nonspecific miRNA (GenePharma) using Lipofectamine 2000.

*DKK2* double-stranded siRNAs were obtained from GenePharma. CHO and HeLa cells were co-transfected with 2 μl of *DKK2* siRNA, 0.2 μg of *pGL3-D3* plasmid using Lipofetamine 2000^TM^ reagent for 24 h. Transfection mixtures were removed and fresh complete DMEM medium was added to each well. Finally, the enzymatic activity of luciferase was then measured with PerkinElmer 2030 Multilabel Reader (PerkinElmer).

### Electrophoretic mobility shift assays

Nuclear protein of pig ovarian follicles was extracted with Nucleoprotein Extraction Kit (Beyotime). Double-stranded oligonucleotides (Sangon) corresponding to the C/EBPβ binding sites of the *DKK2* promoter were synthesized and annealed into double strands. The DNA binding activity of C/EBPβ protein was detected by LightShift^®^ Chemiluminescent EMSA Kit (Pierce). Ten μg ovarian follicle nuclear extract was added to 20 fmol Biotin-labeled double stranded oligonucleotides, 1× binding buffer, 2.5% Glycerol, 5 mM MgCl2, 50 ng Poly (dI•dC), 0.1 mM EDTA and 0.05% NP-40. In addition, control group added 2 pmol unlabeled competitor oligonucleotides. The mixtures were then incubated at 24 °C for 20 min. The reactions were analyzed by electrophoresis in 5.5% polyacrylamide gels in 0.5× TBE buffer at 180 V for 35 min, then were transferred to a nylon membrane. The dried nylon was scanned with GE ImageQuant LAS4000 mini (GE-Healthcare).

### Chromatin immunoprecipitation assay

ChIP assays were performed using the EZ-ChIP™ Kit (Millipore). Briefly, after crosslinking the chromatin with 1% formaldehyde at 37 °C for 10 min and neutralizing with glycine for 5 min at room temperature, PK cells were washed with cold PBS, scraped and collected on ice. Then, cells were harvested, lysed and sonicated. Nuclear lysates were sonicated 20 times for 10 s with 20 min intervals on ice water using a Scientz-IID (Scientz). An equal amount of chromatin was immuno-precipitated at 4 °C overnight with at least 1.5 μg of C/EBPβ (Abcam) and normal mouse IgG (Millipore) antibodies. Immuno-precipitated products were collected after incubation with Protein A+G coated magnetic beads. The beads were washed, and the bound chromatin was eluted in ChIP elution buffer. Then the proteins were digested with Proteinase K for 4 h at 45 °C. The DNA was purified using the AxyPrep PCRCleanup Kit (Axygen).

### qRT-PCR

qRT-PCR was performed on the LightCycler® 480 II (Roche) using iTaq Universal SYBR Green Supermix (Bio-Rad). Primers used in the qRT-PCR were shown in Supplementary Table S3. qRT-PCR conditions consisted of 1 cycle at 94 °C for 3 min, followed by 40 cycles at 94 °C for 20 sec, 60 °C for 20 sec, and 72 °C for 20 sec, with fluorescence acquisition at 74 °C. All PCRs were performed in triplicate and gene expression levels were quantified relatively to the expression of β-actin using Gene Expression Macro software (Bio-Rad) by employing 2–ΔΔCt value^36^.

### Western blotting

Western blotting was performed as reported previously^37^. Five μg proteins were boiled in 5×SDS buffer for 5 min, separated by SDS-PAGE, and transferred to PVDF membranes (Millipore). Then, the membranes were blocked with skim milk and probed with Anti-DKK2 (Santa Cruz). β-actin (Santa Cruz) was used as a loading control. The results were visualized with horseradish peroxidase-conjugated secondary antibodies (Santa Cruz Biotechnology) and enhanced chemiluminescence. All blots were performed in triplicate, and protein expression levels were quantified relative to the expression of β-actin using Image J 1.42q software (Wayne Rasband).

### Cell proliferation analysis

CHO cells were transfected with miRNA mimics using the FuGENE HD transfection reagent (Roche). Cell growth and proliferation were monitored using an xCELLigence RTCA DP instrument (Roche).

### Statistical analysis

Statistical analyses based on two-tailed Student’s *t*-tests were performed using the Statistical Package for the Social Sciences software. Significance was determined at a 95% confidence interval. All data are expressed as the mean ± standard deviation (S.D.).

## Additional Information

**How to cite this article**: Tao, H. *et al.* The transcription factor ccaat/enhancer binding protein β (*C/EBPβ*) and miR-27a regulate the expression of porcine Dickkopf2 (*DKK2*). *Sci. Rep.* **5**, 17972; doi: 10.1038/srep17972 (2015).

## Supplementary Material

Supplementary Information
