# Supplementary material for: The transcription factor ccaat/enhancer binding protein β (C/EBPβ) and miR-27a regulate the expression of porcine Dickkopf2 (DKK2)
Source: Sci Rep. 2015 Dec 11;5:17972. doi: 10.1038/srep17972 (PMC4675968; doi:10.1038/srep17972)
Supplement: Supplementary Information [file srep17972-s1.doc]

**Supplementary Information for:**

**The transcription factor ccaat/enhancer binding protein β (*C/EBPβ*) and miR-27a regulate the expression of porcine Dickkopf2 (*DKK2*)**

**Hu Tao^1, 2^, Lei Wang^1^, Jiawei Zhou^1^, Panfei Pang^1^, Shanzhi Cai^1^, Jianlian Li^1^, Shuqi Mei^2^, Fenge Li^1, 3*^**

^1^Key Laboratory of Pig Genetics and Breeding of Ministry of Agriculture & Key Laboratory of Agricultural Animal Genetics, Breeding and Reproduction of Ministry of Education, Huazhong Agricultural University, Wuhan 430070, PR China

# ^2^Hubei Key Laboratory of Animal Embryo Engineering and Molecular Breeding, Hubei Academy of Agriculture Science, Wuhan, 430064, PR China

^3^The Cooperative Innovation Center for Sustainable Pig Production, Wuhan 430070, PR China

* [lifener@mail.hzau.edu.cn](mailto:lifener@mail.hzau.edu.cn)

**
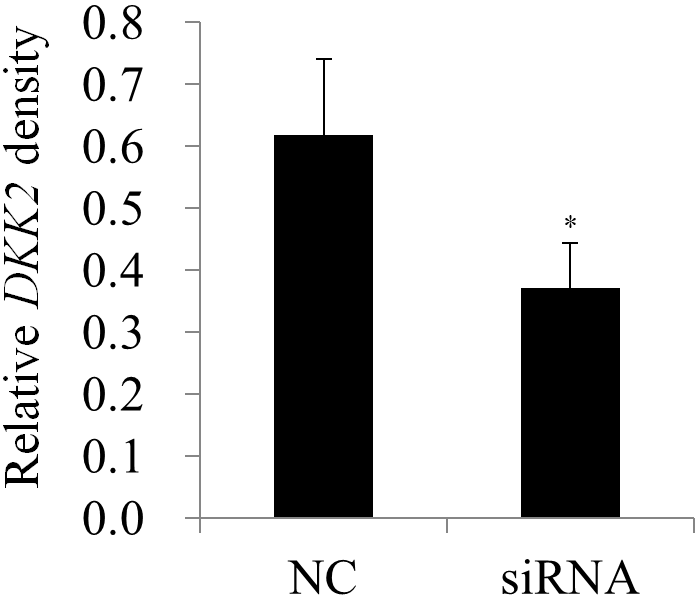
**

Supplementary Figure S1: The gray value of DKK2 protein was determined in Western blotting. All blots were performed in triplicate and protein expression levels were quantified relatively to the expression of β-actin using the Image J 1.42q software (Wayne Rasband). * *P*<0.05.

T/C

○

○

○

○

○

Supplementary Figure S2: Methylation profile of the *DKK2* c.-1130 T>C mutation in the *DKK2* promoter. Bisulfite-treated genomic DNA was amplified and cloned into pMD18-T vector. Five independent clones were sequenced. Open circles indicated un-methylated CpG sites.

Supplementary Table S1: Genotype and allele frequencies of *DKK2* c.-1130 T>C among different pig populations

| Population | N | Genotype | | | Allele frequency (%) | |
| --- | --- | --- | --- | --- | --- | --- |
|  |  | TT | TC | CC | T | C |
| Large White | 274 | 274 | 0 | 0 | 100 | 0 |
| Pietrain | 31 | 31 | 0 | 0 | 100 | 0 |
| Duroc | 29 | 29 | 0 | 0 | 100 | 0 |
| White Duroc | 30 | 30 | 0 | 0 | 100 | 0 |
| Landrace | 21 | 21 | 0 | 0 | 100 | 0 |
| DIV | 129 | 99 | 24 | 6 | 86 | 14 |
| Taihu | 70 | 5 | 22 | 43 | 22.9 | 77.1 |
| Huainan | 20 | 16 | 2 | 2 | 85 | 15 |
| Tongcheng | 37 | 1 | 6 | 30 | 9.5 | 90.5 |
| Hezuo | 7 | 0 | 3 | 4 | 21.4 | 78.6 |

N: number of genotyped pigs. DIV: the 4th dam line of Chinese lean-type new line.

**Supplementary Table S2: Association between *DKK2* c.-1130 T>C and litter size traits in DIV pigs**

| Traits | | Genotype (μ±SE) | | | Effect (μ±SE) | |
| --- | --- | --- | --- | --- | --- | --- |
|  |  |  |  |  |  |  |
|  |  | TT | TC | CC | Additive | Dominance |
| 1st parity | N | 46 | 13 | 3 |  |  |
|  | TNB | 10.61±0.37 | 11.15±0.70 | 11.33±1.45 | -0.36±0.75 | -0.09±0.51 |
|  | NBA | 9.11±0.40 | 9.08±0.74 | 9.00±1.55 | 0.05±0.80 | -0.01±0.55 |
| all parities | N | 387 | 85 | 19 |  |  |
|  | TNB | 10.50±0.19^a^ | 11.81±0.51^b^ | 10.29±0.87^ab^ | 0.01±0.33 | -0.55±0.23* |
|  | NBA | 10.11±0.15^A^ | 11.22±0.31^Bb^ | 9.64±0.67^a^ | 0.31±0.34 | -0.69±0.23** |

N: number of investigated litters. TNB: total number born in litter. NBA: number born alive / litter. a and b: P<0.05. A and B: P<0.01. *: P<0.05. **: P<0.01.

Supplementary Table S3: Primer sequences

| Name | Sequence (5'-3') |
| --- | --- |
| DKK2-PF | CCCCGTTCATTCCTGTTTG |
| DKK2-PR | TTCTCCACGGTCCAATCCT |
| DKK2-PF1 | GCCACCTACTCCTCCAAAGC |
| DKK2-PR1 | CAGCCCTTTTCATGTTTTAGAGC |
| DKK2-QPCR-PF | ATCTGCGGGCACATACCA |
| DKK2-QPCR-PR | CTCCCAACTTCACATTCCTTA |
| DKK2-D1-PF | CTAGCTAGCAGGTATGAAAGCTAGGGAATTCAG |
| DKK2-D2-PF | CTAGCTAGCGGGAGATTGCCTGGGACA |
| DKK2-D3-PF | CTAGCTAGCCCCCGTTCATTCCTGTTTG |
| DKK2-D4-PF | CTAGCTAGCTTTCCTCTAAGGGTGAGTTCTATT |
| DKK2-D5-PF | CTAGCTAGCTCTTGAGGAATTGAGGAGGATT |
| DKK2-D-PR | CCCAAGCTTAGCAATCAAAGGCGAGGC |
| C/EBPb-mut1-PF | GGTACTTTTCCCGAAGAAGCCCCCATCC |
| C/EBPb-mut1-PR | ATGGGGGCTTCTTCGGGAAAAGTACCAG |
| C/EBPb-mut2-PF | CCAGATGCCCATGGTAGCAGAACTTCTA |
| C/EBPb-mut2-PR | CTATAGAAGTTCTGCTACCATGGGCATCT |
| C/EBPb-mut3-PF | CTTCTATAGGGCTGCGAAGATGCTCCTC |
| C/EBPb-mut3-PR | CTAAGAGGAGCATCTTCGCAGCCCTATA |
| C/EBPb-mut4-PF | AGATGAGTTGAAACGCCCATGAA |
| C/EBPb-mut4-PR | CCTTTCATGGGCGTTTCAACTC |
| ChIP -PF | GTACTGTACTGGAAAAGACAAGAA |
| ChIP- PR | TTGTTTAATTTCCTTCCTAGACC |
| DKK2-pcDNA3.1-PF | CTAGCTAGCGCCACCATGGCCGTGTTGATGCGG |
| DKK2-pcDNA3.1-PR | CCCAAGCTTTCATATTTTCTGGCATACATGGA |
| Bcl2-QPCR-PF | TGAGTCGGATCGCAACTTGG |
| Bcl2-QPCR-PR | ATCGGTTGAAGCGTTCCTGG |
| c-MYC-QPCR-PF | GCCAAAAGGTCGGAATCGGGG |
| c-MYC-QPCR-PR | CGCAGCACGTCTTTTTCTGACAC |
| miR-27a-mut-PF | AAAAGCATCGTCAGCAGCTGAATCGTCTGTGTAT |
| miR-27a-mut-PR | TTAAATACACAGACGATTCAGCTGCTGACGATGC |

Note: PF was upstream primer. PR was downstream primer. The part highlighted with grey was enzyme site induced.
